# No Small Worry: Airborne Nanomaterials in the Lab Raise Concerns

**DOI:** 10.1289/ehp.118-a34b

**Published:** 2010-01

**Authors:** Cynthia Washam

**Affiliations:** **Cynthia Washam** writes for *EHP*, *Oncology Times*, and other science and medical publications from South Florida

The use of engineered nanomaterials has grown dramatically over the past decade as the pharmaceutical, electronics, and other industries leverage these materials’ unique physical and chemical properties. In environmental circles, nanomaterials have aroused concern because, even as their use burgeons, their impact on animal and plant life remains largely unknown. Moreover, scientists studying the environmental effects of nanomaterials might unknowingly be putting their own health at risk **[*EHP*** **118:49–54; Johnson et al.]**.

The National Institute for Occupational Safety and Health, which conducts research on workplace safety, has no recommended exposure limit guidelines for nanomaterials, and the Occupational Safety and Health Administration has no permissible exposure limit specific to engineered nanomaterials. However, recent animal toxicology studies suggest nanomaterials may cause specific adverse health effects. For example, carbon nanotubes have been shown to induce inflammation and oxidative stress in animal models.

To assess the magnitude of potential exposure in a laboratory setting, a team of researchers measured the amount of carbonaceous nanomaterials (CNMs) released into the air during routine material handling and processing tasks in standard environmental matrices such as artificial river water. The authors evaluated nanomaterial releases using real-time particle counters and transmission electron microscopy.

The research team found that CNMs became airborne when they were handled and weighed in the lab. Smaller structures, with an aerodynamic diameter of less than 1 μm, scattered more readily than larger particles.

A surprise finding was the substantial release of CNMs during sonication, a common laboratory process used to break apart agglomerates of nanomaterials into aqueous dispersions. Sonication produced a CNM-containing mist that could be inhaled by workers or that could leave CNMs on laboratory surfaces after the water evaporated. The extent of release during sonication was increased when natural organic matter was added to the solution, as is often done to simulate conditions in the environment. Hydrophobic CNMs exhibited higher airborne particle number concentrations during handling than during sonication, whereas hydrophilic CNMs exhibited the opposite trend.

These findings contradict the belief that risks of exposure are minimized when working with nanomaterials in liquid suspensions. The authors believe this field case study is the first to demonstrate the release of CNMs during sonication and also the first to detail nanomaterial release in an environmental laboratory. They caution that more robust statistically based experimental research is needed to evaluate CNM exposure among laboratory workers. Until then, they urge researchers working with nanomaterials to use appropriate personal protective equipment in the laboratory and to adopt adequate engineering controls to minimize their exposure.

